# American Horseflesh in European Food-markets

**Published:** 1895-05

**Authors:** 


### SELECTIONS.

THE United States Consul at Brunswick, Germany, in a recent report to our Government, has this to say on the subject of horseflesh as an article of diet in Germany: That it is the chief meat of a large percentage of the poorer classes in that country; that it is sold in all the large cities, where numerous shops exist for its sale, and the quantity consumed in these districts equals that of beef or mutton; that the present high price prevailing of forty to fifty dollars per head opens the market for the great surplus still glutting the American horse market, and for which there promises no great relief for a year or two. He advises American ranch owners, stockmen, and meat packers to give the matter immediate consideration, specially so since the exclusion of American cattle from German ports. Horseflesh is offered for sale in smoked and salted forms of preparation. It could be readily prepared on the other side if shipped alive on the hoof.

This is a matter that should command thorough consideration by our American raisers of horses, as the present extremely low prices are impoverishing the producers in the West, and have bankrupted many prosperous farmers. No horse can be raised and sold at four to five years of age at fifty dollars per head, as the food, care, and losses entailed in horse-breeding farms make it impossible. To-day our Eastern markets are glutted with a class of horses which, after the added expense of transportation, etc., range from fifty to eighty dollars in price. Now many of these horses are at two years of age but little less in size and weight than at four, at which age it is the earliest that they can be offered for sale for use. Now when used for beef there would be a saving of two years' cost of keep, lessened losses in all directions, and at forty dollars per head would pay a fair return for raising. It will be some time before our American people will overcome that present repugnance against horseflesh as an article of food, though it will every day grow to be a stronger factor in the regulation and control of the beef market, and the present opportunity would be a good one to grasp and a great outlet for the over-production now existing in the horse-raising centres of our land.

